# Orientation and Oviposition by Female *Plodia interpunctella* (Lepidoptera: Pyralidae) in Response to Volatiles from Varieties of Peanuts

**DOI:** 10.3390/insects16111145

**Published:** 2025-11-08

**Authors:** Xi Zhu, Dianxuan Wang, Fangfang Zeng, Liang Chen, Chen Wang, Sijia Shang, Zixin Guo

**Affiliations:** Grain Storage and Logistics National Engineering Research Center, National Grain Industry (Storage Insect Pest Control) Technology Innovation Center, Henan University of Technology, Zhengzhou 450001, China; zhu_xi537@163.com (X.Z.); zengfangfang@haut.edu.cn (F.Z.); 18623718386@163.com (L.C.); 18638764239@163.com (C.W.); shangsijia2024@163.com (S.S.); 18836928631@163.com (Z.G.)

**Keywords:** stored-product pest, peanut volatiles, behavioral response

## Abstract

Some special volatile organic compounds (VOCs) that significantly induce female oviposition preferences may be utilized to disrupt oviposition behavior and to enhance trapping strategies; such approaches offer a promising avenue for reducing insect infestations in stored commodities. Based on the significant differences in the oviposition preference of *P. interpunctella* among six normal-oleic varieties (NOPs), the key VOCs involved were further explored. Seventeen VOCs that may contribute the oviposition preference and that exhibited a high content in the peanut varieties were measured through electroantennogram (EAG) response measurements of female moths. The VOCs that produced significant EAG responses by the females were further assayed for behavioral responses by the Y-tube olfactometer method, wind tunnel tests, and a multiple-choice device for female oviposition. Heptanal, acetophenone, nonanal, hexanal, benzaldehyde, octanal, hexanoic acid, decanal, phenylacetaldehyde, and 1-octen-3-ol from peanuts elicited strong antennal EAG responses. These VOCs (especially heptanal, nonanal, hexanal, octanal, and decanal) attracted more females in both Y-tube olfactometer and wind tunnel assays and increased oviposition rates in oviposition tests. The results indicate that heptanal, decanal, octanal, nonanal, and hexanal may be utilized to develop oviposition attractants for female moths further.

## Simple Summary

The Indian meal moth, the *Plodia interpunctella* (Hübner, 1813) (Lepidoptera: Pyralidae), is a globally widespread stored-product pest. Female *P. interpunctella* show a stronger oviposition preference for normal-oleic peanuts over high-oleic peanuts, maize, wheat, and paddy rice. We hypothesize that this preference for peanuts over grains is mediated by plant volatiles. In this study, we collected the volatile compounds from peanuts by dynamic headspace adsorption, selected seventeen compounds that are commonly found in most peanut varieties and have relatively high contents, and evaluated the electrophysiological and behavioral responses to the *P. interpunctella*. Aldehydes, especially heptanal, nonanal, hexanal, octanal, and decanal, have a significant attraction to moths. Our results provide insight into the chemical cues used by *P. interpunctella* females to locate the host. These compounds could potentially be developed as novel tools for monitoring and controlling *P. interpunctella*.

## 1. Introduction

Postharvest loss encompasses quantity and quality losses, reducing the economic value of stored products and making them unsuitable for human consumption [1]. Stored-product insects can cause huge losses, threatening food security and sustainability, thereby exacerbating hunger and increasing the usage of agricultural resources. The use of chemically synthesized insecticides, including toxic fumigants, has been the most effective pathway of combating stored-product insect pests, especially through fumigation in sealed enclosures. Controlled atmosphere technology has also undergone rapid development [2]. The management of stored-product pests has to shift to a more integrated approach, since in many cases unsuitable conditions for insecticide application, fumigation, or controlled atmosphere treatments are present [3]. At the commodity storage warehousing and shipping level, managers can rely on chemical treatments, aerosols, or spot treatments to control pest populations [4]. These situations, such as retail or grocery environment, have to abandon the use of insecticides, fumigation, or atmosphere control due to a lack of airtightness or safety considerations [5].

One of the promising and expanding biorational insect pest control strategies is the application of trapping methods, which have been rapidly developing over the last several decades [6]. A well-established example is mating disruption, a strategy that prevents successful mating by inundating the environment with synthetic pheromones, thereby reducing egg loads and larval populations [7,8,9]. This approach targets male behavior by saturating the environment with pheromones, disrupting males’ ability to locate and mate with females. Mating disruption has been applied in stored product protection [10,11,12,13] and is commercially available in facilities such as flour mills, warehouses, and manufacturing plants [11,14]. In addition to pheromone-based disruption, exploiting female oviposition preferences through volatile organic compounds (VOCs) represents a parallel, biorational strategy [15,16]. Previous studies have shown that stored-product pests including the sawtoothed grain beetle, *Oryzaephilus surinamensis* (Coleoptera: Silvanidae), were attracted to nonanal, dodecane, and β-caryophyllene released from a mixture of six food sources including rolled oats [17]. The rice weevil, *Sitophilus oryzae* (Coleoptera: Curculionidae), was attracted to nonanal emitted from rice grains [18]. The maize weevil, *Sitophilus zeamais* (Coleoptera: Curculionidae), showed strong orientation responses to volatiles from maize and wheat seeds but was significantly repelled by odors from cubeb pepper and ginger rhizomes [19]. In addition, the lesser grain borer, *Rhyzopertha dominica* (Coleoptera: Bostrichidae), exhibited a stronger attraction to wheat than to soybeans, maize, and other cereals, and 1-hexanol was the most abundant VOC in wheat grains, playing a key role [20]. Utilizing female oviposition preferences to disrupt their orientation toward traps instead of infesting stored commodities may be a pathway to be exploited.

The Indian meal moth, *Plodia interpunctella* (Hübner, 1813) (Lepidoptera: Pyralidae), is a major pest of stored products worldwide, reported in 48 countries and known to infest 179 commodities, including grains, confectionery, nuts, dried fruits, and medicinal materials [21]. This pest causes substantial economic losses and quality deterioration in both agriculture and the food industry [22]. Its life cycle includes egg, larval, pupal, and adult stages, with larvae being the most destructive. Larvae not only feed but also spin webs that contaminate food, often making products unsuitable for consumption [23]. The oviposition behavior of adult females plays a key role in population growth. Previous studies have shown that fungus-infected wheat releases the unique volatile compound 3-methyl-1-butanol, which may act as a quality cue for mated females seeking suitable oviposition sites [24]. In addition, compared with wheat, maize, and rice, *P. interpunctella* larvae display a stronger feeding preference for peanuts, while adults also show a higher tendency to oviposit on peanuts [25].

The *P. interpunctella* is a major pest in stored peanuts [21]. Studies have shown significant differences in its oviposition preferences among peanut varieties. Compared with high-oleic peanuts (HOPs), which contain more than 80% oleic acid, *P. interpunctella* shows a stronger tendency to oviposit on normal-oleic peanuts (NOPs) [26]. Oleic acid itself is a non-volatile fatty acid, but its concentration may be associated with the type and quantity of volatiles released by peanuts [27]. We therefore hypothesize that NOPs may emit specific VOCs that induce oviposition in female *P. interpunctella*. If identified and developed as attractants, such VOCs could be used for monitoring or trapping females, thereby reducing pest infestations in stored peanuts.

For the exploring a pathway of the integrated pest management of *P. interpunctella*, the objectives here include (1) identifying and quantifying VOCs; (2) exploring the relationship between characterizing antennal electrophysiological responses and behavioral responses; (3) evaluating the attractiveness in wind tunnel assays; and (4) comparing oviposition among the special VOCs in same environment. The results may be valuable information for developing attractant VOCs to disturb female oviposition choices of stored-product moths.

## 2. Materials and Methods

### 2.1. Insects

The tested population of *P. interpunctella* was collected from a grain depot at Zhengzhou, Henan Province, China, and was reared continuously in the laboratory for approximately two years (>12 generations) with rolled oats, corn flour, and yeast (19:19:2 in proportion) at 28 ± 1 °C, 75 ± 5% r.h., and 16:8 (L:D) [26]. The gender of the moths was distinguished by a dark spot on abdomen of male final-instar larvae [28]. The female and male were kept separately and individually before mating. Two-day-old mated females were used for electroantennogram assays and behavior experiments.

### 2.2. Peanuts

The varieties of peanut were Yuhanghua 1, Yuanza 9847, Wanhua 2, Huayu 16, Fenghua 1, and Yuhua 9719. They were harvested in the same year and dried under natural sunlight for 3–5 days and stored at 4 °C for two weeks and then at −20 °C for one week to eliminate insects. Before analysis, the peanut samples were kept at 25 ± 1 °C one week before the measurements. The chemical characteristics of each tested peanut were determined by peanut quality rapid analyzer (Peanut 1.0, Beijing Kaiyuan Hongyu Technology Co., Ltd., Beijing, China) (Table 1). Each variety was tested in triplicate.

### 2.3. VOC Collection from Peanut Samples

The dynamic headspace adsorption method was used to determine the peanut volatiles [26], and a dynamic headspace adsorption instrument (Hongyi Instrument Company, Wuhan, China) was utilized. The device consisted of a custom-made glass jar (12.6 cm in diameter and 20 cm in height). The jar lid was designed with two connectors, one for connecting purified air and another for attaching an adsorption column, and a glass tube (0.9 cm in diameter and 18 cm in height) filled with 100 mg of Porapak™Q adsorbent (Waters Company, Milford, MA, USA) or venting to ambient air. The purified air stream was controlled and regulated using an air pump (ACO-002 electromagnetic air pump, Sensen, Zhengzhou, China), a filter containing activated carbon, a vial containing silica gel, and a flowmeter before entering the jar.

A total of 200 g of peanut samples was sealed inside the glass jar, and volatiles were collected by passing purified air (300 mL/min) through the sample for 12 h. Volatile compounds trapped on the adsorption column were then eluted with 1 mL of n-hexane (GC grade, purity ≥ 99%, Shanghai Macklin Biochemical Technology Co., Ltd., Shanghai, China) and concentrated to 0.20 mL using nitrogen gas, stored in 2 mL GC vials, and preserved at −80 °C for subsequent analysis using gas chromatography–mass spectrometry (GC-MS). To correct for impurities, blank controls were run concurrently with the peanut samples by collecting air volatiles in the absence of peanuts under identical conditions. To assess potential overlap between components of the paraffin oil carrier and peanut-derived VOCs, paraffin oil was dissolved in n-hexane (10-fold dilution) and analyzed using the same experimental procedures as those used for the peanut VOC determinations.

### 2.4. VOCs Analyzed by GC-MS

The chemical analysis was performed using a GC-MS-QP 2010 Ultra (Shimadzu Co., Ltd., Kyoto, Japan) equipped with a HP-5 MS capillary column (30 m × 0.25 mm × 0.25 μm) [29]. The injection was carried out in splitless mode, with 1 μL of the extract containing 2 ng of 2-octanol (internal standard, purity ≥ 99.50%, Aladdin, Shanghai Aladdin Biochemical Technology Co., Ltd., Shanghai, China) injected. The temperature program was as follows: the initial temperature was held at 35 °C for 4 min, followed by an increase at a rate of 4 °C/min to 125 °C and held for 3 min, then further increased at 4 °C/min to 165 °C and held for 3 min, and finally, it was ramped at 10 °C/min to 250 °C and held for 3 min. The injector temperature was 250 °C, and helium was used as the carrier gas at a flow rate of 1.0 mL/min.

The mass spectrometer conditions were as follows: the injector temperature was 250 °C, the interface temperature was 250 °C, and the ion source was operated in electron ionization (EI) mode at 230 °C with an electron energy of 70 eV. Data were acquired in full scan mode across a mass range of 50–550 *m*/*z*. Volatile compounds were identified using the NIST-17 (National Institute of Standards and Technology, Gaithersburg, MD, USA) and Wiley 275 libraries (John Wiley & Sons, Inc., Hoboken, NJ, USA) (match ≥ 95%). An additional criterion for peak assignment was consistency between the temperature-programmed retention indices (RIs) obtained and those recorded in the NIST-17 database. The RIs were calculated based on the retention time of a sequence of alkanes C8–C40 (Merck KGaA, Darmstadt, Germany) for each selected VOC. RI^a^ was determined on a HP-5 MS column using a homologous series of n-alkanes (C8–C40). RI^b^ was obtained from the literature for compound whose identity was established based on comparison of retention time and mass spectra data with authentic standard. Each variety was analyzed in three biological replicates.

### 2.5. Chemicals

The tested chemicals, simulated with VOCs from the peanuts, including decanal (97%), heptanal (97%), hexanal (99%), octanal (99%), benzaldehyde (98.5%), phenylacetaldehyde (95%), acetophenone (99%), dodecane (99%), tetradecane (98%), hexadecane (98%), heptadecane (95%), decane (99%), hexanol (98%), hexanoic acid (99%), 1-octen-3-ol (98%), and limonene (95%), were purchased from Shanghai Macklin Biochemical Technology Co., Ltd. (Shanghai, China). Nonanal (95%) was purchased from J&K Scientific Co., Ltd. (Beijing, China). These compounds were diluted with liquid paraffin (Macklin, Shanghai, China) to different concentrations (0.1, 1, 10, and 100 μg/μL) for the assay.

### 2.6. Electroantennogram Bioassay

The electroantennograms (EAG) were conducted to determine whether the chemicals in peanut volatiles could stimulate the antennae of mated females [30]. Each chemical was diluted in paraffin oil to prepare 0.1, 1, 10, and 100 μg/μL solutions, with 10 μL of paraffin oil as a control. The dissected antennae were fixed securely between the fork-shaped metal electrodes of the EAG probe (PRG-3 EAG Combiprobe, Syntech, Kirchzarten, Germany) using conductive gel (Spectra 360, ParkerLab, Fairfield, NJ, USA), ensuring a 1 cm gap between the antennae and the odor delivery tube. For testing, 10 μL of solution was positioned on a filter paper strip (0.5 cm × 3 cm, and the solvent was allowed to evaporate from the filter paper for 30 s. Then, filter paper was placed inside a glass Pasteur pipet (0.7 cm in diameter by 23 cm in length), and its tip was inserted into a small hole in the mixing tube. The stimulus controller (CS-05, Syntech) provided a continuous airflow that was filtered through activated charcoal and humidified, with a flow rate of 300 mL/min. The stimulus duration was 1 s, with a 60 s interval allowed between each stimulation to allow for recovery of antennal sensitivity. Paraffin oil was used as a control before and after the volatile stimulation reaction. Analog signals were analyzed using EAG software version 2.6 (Syntech, Kirchzarten, Germany). Six antennae were tested for every concentration of each compound. The calculation formula for the EAG response of each sample is

*r*EAG = EAG_x_ − (EAG_ck1_ + EAG_ck2_)/2 (1)

where *r*EAG indicates relative EAG response; EAGx is the amplitude (mV) of the EAG response to a tested sample; and EAG_ck_ are responses to the first and the second control stimuli. The absolute EAG response to the paraffin oil control stimuli was consistently low across all experiments, with a mean value of 0.037 ± 0.003 mA.

### 2.7. Y-Tube Olfactometer Bioassays

Ten individual compounds (1-octen-3-ol, hexanal, heptanal, octanal, nonanal, decanal, benzaldehyde, acetophenone, phenylacetaldehyde, and hexanoic acid) that evoked obvious EAG responses were selected to examine the behavior of mated females using a Y-tube olfactometer [31]. The Y-tube olfactometer had a 4 cm inner diameter, with a 30 cm common tube and two arms that were extended 20 cm at 75°. Before bioassays, all glassware was cleaned with absolute alcohol and heated at 180 °C for 3 h. The test solutions were dissolved in paraffin oil in different concentrations (0.1, 1, 10, and 100 μg/μL). A steady airflow (300 mL/min), filtered through activated charcoal and humidified, was pumped into each source container using an atmospheric sampling instrument. A filter paper strip (3 cm × 0.5 cm) was placed in each of the two arms of the Y-shaped olfactometer; 10 μL of test solution was added to one arm, and an equal volume of paraffin oil was added to the other arm. The solvent on the filter paper strip was evaporated for 30 s. A “choice” was recorded when the test insect crossed 10 cm of one of the two olfactory arms within 5 min and stayed there for more than 10 s; otherwise, it was artificially recorded as “no choice”. Sixty females were tested individually for each VOC concentration. During the assays, the Y-tube olfactometer was cleaned with anhydrous ethanol and distilled water and dried after every five females to eliminate residual odors. The Y-tube was reversed after five moth tests to avoid positional bias.

### 2.8. Wind Tunnel Bioassays

**Figure 1:**
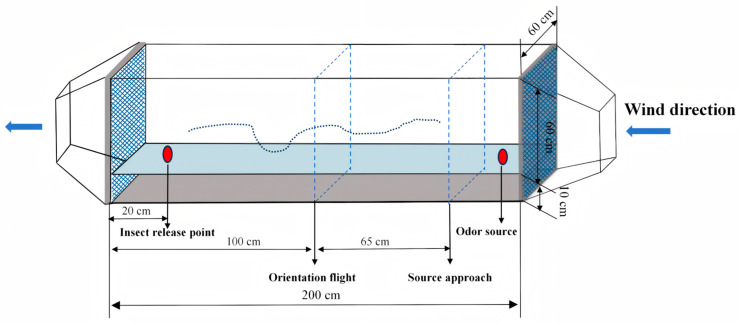
Schematic illustration of wind tunnel used to test *P. interpunctella* attraction to odors. The wind direction is indicated by the blue arrows. The insect release point, odor source placement point, orientation flight zone, and source approach zone are shown in the figure. As insects fly upwind from the release point, they are able to perceive the odor plume and respond accordingly.

The wind tunnel (200 cm × 60 cm × 70 cm), made of transparent Perspex acrylic, was vertically divided by a transparent partition into an upper (60 cm) compartment for clean odor environment experiments and a lower (10 cm) compartment for simulating background grain odors when the partition was removed (Figure 1). Fans (FWY-250-2, Zhejiang Yishen Electromechanical Co., Ltd., Taizhou, China) were installed at each end of the tunnel, connected to the air supply and exhaust systems. The outside air was blown into the tunnel by a fan through the activated carbon filter and 100-mesh metal screen, and the air out of the tunnel passed through the 100-mesh metal mesh screen and the activated carbon filter and then was discharged to the outside through the exhaust system. The wind speed was set at 0.3 m/s, and the wind speed at each point of the wind tunnel was measured by a hot-film anemometer (AR866, Dongguan Xintai Instruments & Meters Co., Ltd., Dongguan, China). The experiments were conducted in a dark room, with all flight tests performed two hours before the dark phase. The room was kept at 25 ± 2 °C and 60–70% r.h. Before each test, the clean air filtered with activated carbon was passed through a wind tunnel for 15 min to decontaminate any potential odors.

The behavioral responses of mated females to the compounds at the selected optimal concentrations were studied in a wind tunnel [32]. A 2-day-old mated female was placed in a glass tube (3 cm diameter, 10 cm height) closed at both ends with a curtain net and placed on a stand 20 cm downwind and 20 cm high above the ground. Then, 20 μL of volatile solution was added to a rubber septum. The rubber septum was then placed on a support 20 cm upwind in the wind tunnel and 20 cm above the ground. The behavior of moth was observed and recorded as non-responsive, take-off, orientation flight (taking upwind flight, over 1/2 of the total length of the wind tunnel), source approach (15 cm from the source of the odor), and landing. Each moth was tested for 3 min, and each treatment was tested in three independent replicates, with 20 mated females per replicate (total of 60 females per treatment).

### 2.9. Oviposition Bioassay

**Figure 2:**
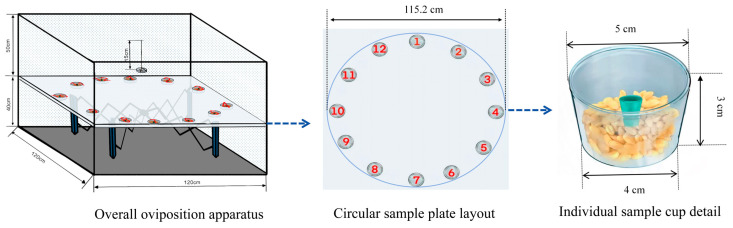
Schematic diagram of the multiple-choice oviposition apparatus used for *P. interpunctella* females, consisting of the overall oviposition apparatus (120 cm × 120 cm × 90 cm, two-layer structure), a circular sample plate layout (12 numbered positions, 115.2 cm in diameter), and the detail of an individual sample cup (5 cm in top diameter, 4 cm in bottom diameter, and 3 cm in height, containing a wheat substrate and a rubber stopper).

The oviposition assay of *P. interpunctella* females was carried out in a multiple-choice oviposition apparatus (120 cm × 120 cm × 90 cm) (Figure 2). A sample plate, containing 12 cups for the flavor source, and a hanging Petri dish for the mating moths were positioned at a height of 40 cm from the bottom of the unit. In each sample plate, 8 treated flavor source cups were placed alternately with 2 control cups and 2 blank cups. Twelve cups were set aside with 10 g of wheat of equal mass. The treated wheat was fixed with a rubber stopper with 400 μL of each compound (all at a concentration of 10 µg/µL: heptanal, nonanal, hexanal, decanal, octanal, 1-octen-3-ol, and acetophenone, as well as hexanoic acid at 1 µg/µL). The control whole wheat was fixed with a rubber stopper with 400 μL of paraffin oil. Blank group of wheat was fixed with a blank rubber stopper. Three mating pairs of female and male adults of *P. interpunctella* were released in each apparatus, and egg numbers on each cup were counted after 72 h. Each treatment was replicated nine times.

### 2.10. Statistical Analysis

Before analysis, the Shapiro–Wilk test and Levene’s test were used to check data normality and homogeneity of variances, respectively. Relative EAG response data, behavioral categories of *P. interpunctella* (non-response, take-off, orientation flight, source approach, and landing), and oviposition data were analyzed by one-way analysis of variance (ANOVA), followed by Tukey’s test (*p* < 0.05). Difference in Y-tube olfactometer bioassay data was analyzed by the chi-squared test. All data analyses were performed using SPSS version 27.0 (Chicago, IL, USA).

## 3. Results

### 3.1. Special VOCs in Tested Peanut Varieties

**Figure 3:**
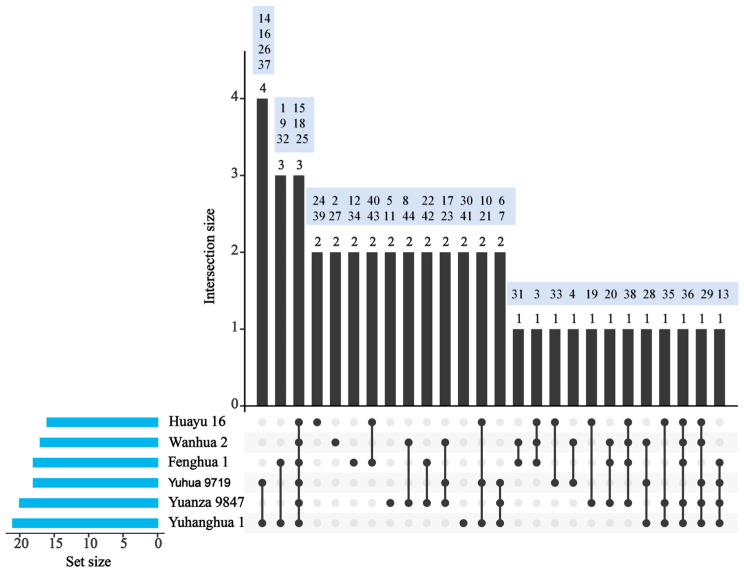
Upset plot illustrating the distribution and intersection of volatile compounds among the six peanut varieties. The horizontal blue bars on the left (Set Size) indicate the total number of volatiles detected in each variety. The vertical black bars on the top (Intersection Size) represent the number of volatiles in each corresponding intersection shown in the matrix below. In the matrix, single dots denote volatiles unique to a single variety, whereas dots connected by a line denote volatiles shared by the indicated varieties. The numbers in the blue boxes correspond to the serial numbers of the VOCs listed in Table 2.

The number of VOCs analyzed from the tested peanuts by the GC-MS method was 21 for Yuhanghua 1, 18 for Yuhua 9719, 18 for Fenghua 1, 16 for Huayu 16, 17 for Wanhua 2, and 20 for Yuanza 9847 (Figure 3 and Table 2). Decane, limonene, and nonanal were detected in all six tested varieties. Unique components were found in Yuhanghua 1(4-methyl-1-undecene and 2,6-dimethyl-heptadecane), Fenghua 1(1,1′-(1,4-phenylene)bis-Ethanone and 1-methyl-2-propyl-cyclohexane), Huayu 16(2-methyl-decane and 6-methyl-octadecane), Wanhua 2(butyl acetate and 2-decen-1-ol), and Yuanza 9847 (1,2,3-trimethyl-Benzene and 2,5-dimethyl-heptane). The paraffin oil was analyzed under the same GC-MS conditions as the peanut volatile compounds, and none of the 44 peanut-derived volatile compounds listed in Table 2 were detected. Seventeen VOCs were detected in most varieties and were present at relatively high abundances. These compounds were selected for further antennal response analysis with the EAG method.

### 3.2. Electrophysiological Responses of P. interpunctella Females to Seventeen VOCs

**Figure 4:**
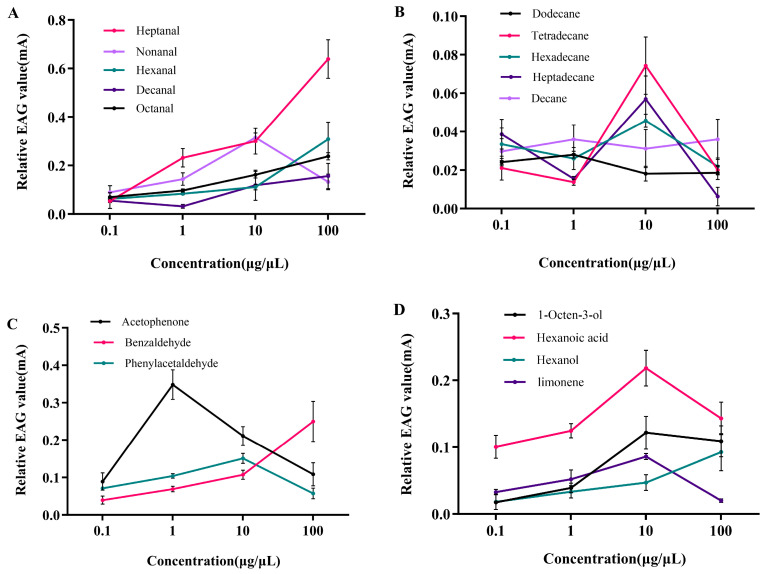
EAG responses (mean ± SE, *N* = 6) of mated *P. interpunctella* females to VOCs at four concentrations. (**A**) Aldehyde compound, (**B**) alkane compound, (**C**) aromatic compound, and (**D**) other compounds.

**Figure 5:**
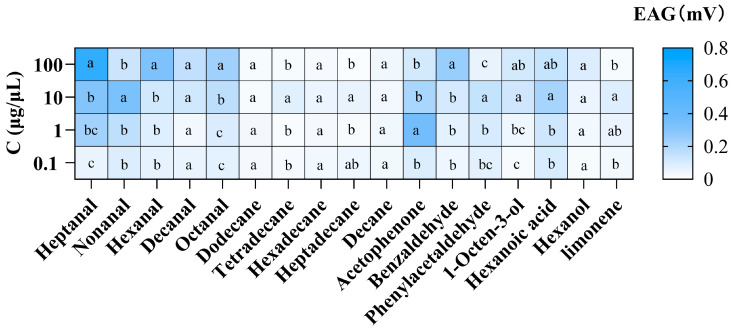
EAG response differences in mated female *P. interpunctella* to various concentrations of VOCs. The color-coded heatmap represents the average EAG response (in mV) to each compound at different concentrations. The color bar on the right indicates the amplitude of the EAG response. Data in the same row with different lowercase letters are significantly different (*p* < 0.05, one-way ANOVA, followed by Tukey’s test).

The EAG values of the female antennae to aldehyde, alkane, aromatic, alcohol, acid, and alkene are shown in Figure 4. The EAG response differences among different concentrations for the same VOC are given in Figure 5. The highest EAG response was elicited by 100 μg/μL of heptanal, with an EAG value of 0.64 ± 0.08 mV, followed by 1 μg/μL of acetophenone (0.35 ± 0.04 mV), 10 μg/μL of nonanal (0.32 ± 0.02 mV), 100 μg/μL of hexanal (0.31 ± 0.07 mV), and 100 μg/μL of benzaldehyde (0.25 ± 0.05 mV). Compounds such as octanal, hexanoic acid, decanal, phenylacetaldehyde, and 1-octen-3-ol showed moderate responses ranging from 0.10 to 0.23 mV. The other seven compounds, including hexanol, limonene, tetradecane, heptadecane, hexadecane, decane, and dodecane, elicited very weak responses or no response (an EAG value < 0.10 mV). The VOCs that elicited an EAG response above a threshold of 0.10 mV were selected for evaluation in behavioral assays.

### 3.3. Behavior Response of P. interpunctella Female to VOCs in Y-Tube Olfactometer Assay

**Figure 6:**
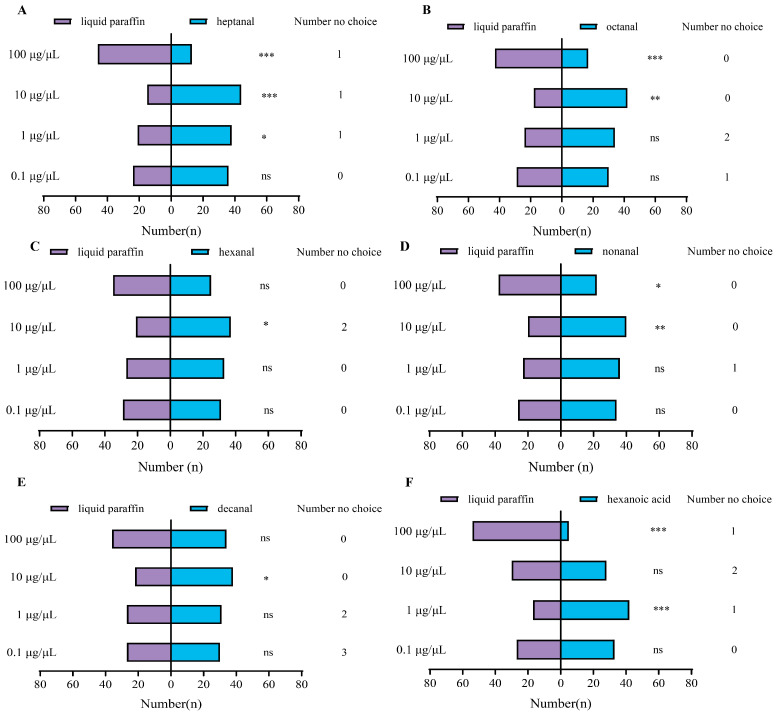

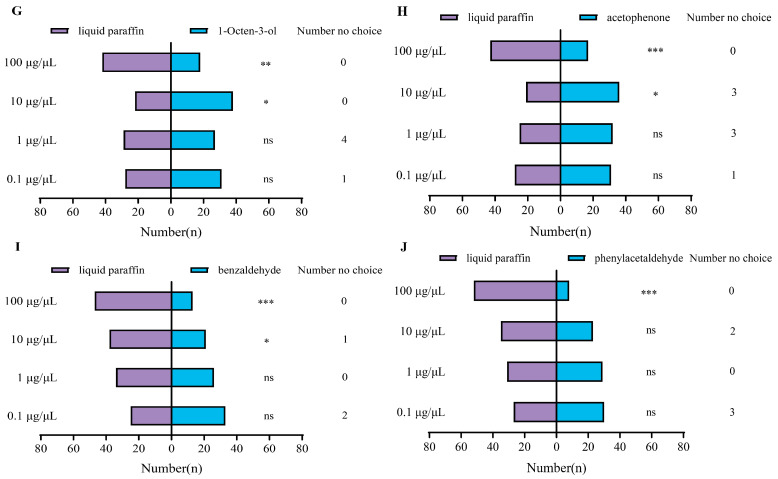
Behavioral responses of mated *P. interpunctella* females to the compounds at different concentrations (*N* = 60). (**A**) Heptanal, (**B**) octanal, (**C**) hexanal, (**D**) nonanal, (**E**) decanal, (**F**) hexanoic acid, (**G**) 1-octen-3-ol, (**H**) acetophenone, (**I**) benzaldehyde, and (**J**) phenylacetaldehyde. * *p* < 0.05; ** *p* < 0.01; *** *p* < 0.001; and “ns” indicates no significant difference (χ^2^-test).

Figure 6 indicates that the mated females are significantly attracted to heptanal (χ^2^ = 14.254, *p* < 0.001), octanal (χ^2^ = 9.600, *p* < 0.01), hexanal (χ^2^ = 4.414, *p* < 0.05), nonanal (χ^2^ = 6.667, *p* = 0.01), decanal (χ^2^ = 4.267, *p* < 0.05), 1-octen-3-ol (χ^2^ = 4.267, *p* < 0.05), and acetophenone (χ^2^ = 3.947, *p* < 0.05) at 10 μg/μL. No significant responses were recorded at 0.1 and 1 μg/μL of all tested VOC concentrations, excepted for heptanal (χ^2^ = 4.898, *p* < 0.05) and hexanoic acid (χ^2^ = 10.593, *p* = 0.001) at 1 μg/μL. The females showed no attractive response to benzaldehyde and phenylacetaldehyde. Notably, all volatiles exhibited significant repellent effects on *P. interpunctella* at a concentration of 100 μg/μL.

### 3.4. Behavior Response of the Females to VOCs in Wind Tunnel Measurement

**Figure 7:**
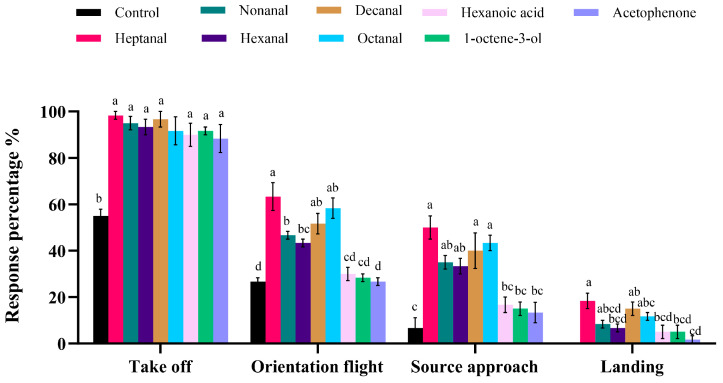
The behavioral responses of *P. interpunctella* female moths to specific VOCs and a control (paraffin oil). The bar charts show the percentage of females that exhibited take-off, orientation flight, source approach, and landing behaviors in response to each VOC. Different letters on the top of each bar indicate significant differences between the treatments for each behavior response (one-way ANOVA, followed by Tukey’s test, *p* < 0.05).

*P. interpunctella* exhibited higher take-off, orientation flight, source approach, and landing responses to the eight tested volatiles than to the control (Figure 7). During the orientation flight and source approach, females responded most strongly to heptanal, then octanal and decanal. Responses to hexanoic acid, 1-octen-3-ol, and acetophenone were comparatively low and did not differ significantly from the control. Heptanal produced the highest landing rate, while decanal and octanal showed intermediate but positive landing responses.

### 3.5. The Oviposition Laid on Wheat with Different VOCs

**Figure 8:**
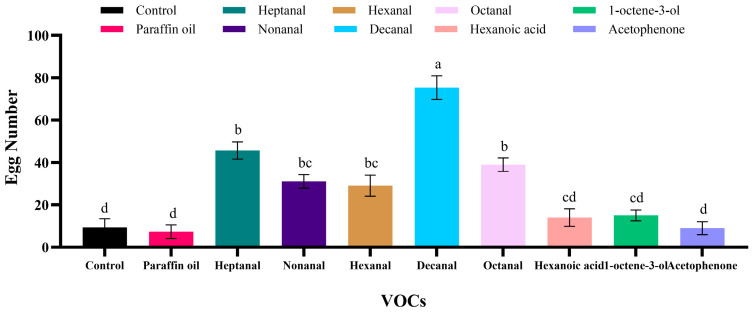
Oviposition assays of female *P. interpunctella* in response to eight peanut-derived volatiles. The bar chart shows the oviposition (mean ± SE) laid by female moths exposed to each compound and the control groups (control and paraffin oil). The tested volatiles were heptanal, nonanal, hexanal, decanal, octanal, hexanoic acid, 1-octen-3-ol, and acetophenone. Different lowercase letters above the bars indicate significant differences among the treatments (one-way ANOVA, followed by Tukey’s test, *p* < 0.05).

In this study, among the eight tested volatiles, the number of eggs in the wheat containing decanal, heptanal, octanal, nonanal, and hexanal was significantly higher than the control (decanal: *p* < 0.001; heptanal: *p* < 0.001; octanal: *p* < 0.001; nonanal: *p* = 0.023; hexanal: *p* = 0.048, Figure 8). Compared to the control, there was no significant oviposition response from the female *P. interpunctella* to hexanoic acid, 1-octen-3-ol, or acetophenone (hexanoic acid: *p* = 0.997; 1-octen-3-ol: *p* = 0.987; acetophenone: *p* = 1.000). There was no significant difference in the number of eggs between the wheat treated with paraffin oil and that treated with an empty rubber stopper (control) (*p* = 1.000).

## 4. Discussion

VOCs emitted by stored commodities can act as attractants or repellents and are increasingly considered promising tools for the integrated pest management of stored-product pests [25,33,34,35]. However, it is important to note that not all volatiles released from storage are of biological or ecological significance. Peanuts are considered the preferred host for *P. interpunctella* [25]. In the present study, we selected 17 volatiles from peanut VOCs and screened for volatiles that elicited significant attractant behavior in *P. interunctella* females. These compounds may be used as potential attractants and provide methods for monitoring and controlling storage insects.

The headspace volatiles of peanuts were analyzed using GC-MS, and the identified compounds were mainly alcohols, aldehydes, ketones, terpenes, and alkanes. Although the most abundant compound varied by varieties, a core set of volatiles was common across varieties. The presence of such conserved components suggests they may provide general host cues for *P. interpunctella*, whereas variety-specific compounds could modulate fine-scale oviposition preferences. These results are consistent with previous reports that identified hexanal, nonanal, and decanal among peanut volatiles [26,36], and they support the idea that a combination of ubiquitous and cultivar-unique VOCs determines moth attraction and oviposition behavior. From an applied perspective, ubiquitous compounds are promising candidates for broad-spectrum lures, whereas variety-specific markers may help explain differential infestation levels and guide variety-specific management strategies.

The EAG screening process helps reduce the number of VOCs tested, allowing for the identification of promising bioactive compounds and screening effective substances for further behavioral testing [37,38]. The EAG response results indicated that all tested aldehydes had antennal sensitivity, which suggests that *P. interpunctella* females can recognize these compounds at the peripheral olfactory level. This might be due to the fact that the olfactory receptor neurons (ORNs) in the sensilla trichodea, which are the primary receptors for host volatiles in female *P. interpunctella*, exhibit a significant stress response to aldehyde volatiles [39,40]. Female *P. interpunctella* were found to exhibit antennal sensitivity to hexanal, octanal, heptanal, nonanal, and decanal [39], which is consistent with our findings. Since the EAG response represents the sum of the reactions from the entire antenna’s sensilla, the less pronounced EAG response to alkanes may be due to the generally lower frequency of these five alkane-sensitive sensilla at the antennae [41].

It is important to note that the ability of herbivorous insects to perceive chemicals through olfactory receptors does not necessarily mean that these chemical signals directly influence their behavioral responses (attraction or repulsion) [30,42]. Therefore, behavioral experiments are necessary to determine the relationship between these olfactory signals and insect behavior during the host orientation. In the Y-tube olfactometer behavioral bioassays, 10 µg/µL of heptanal exhibited strong attraction effects on mated *P. interpunctella* females, followed by hexanoic acid, nonanal, and octanal. It is noteworthy that at a concentration of 100 µg/µL, all compounds demonstrated repellent effects on the females. The concentrations of volatiles used in the behavioral assays of this study were higher than those naturally present in peanuts. However, the selected concentrations were based on commonly applied ranges in previous studies on stored-product insects. The purpose was to effectively elicit and quantify insect behavioral responses under laboratory conditions, thereby establishing a clear dose–response relationship to identify bioactive compounds and determine their effective ranges. Similarly, nonanal was discovered to be attractive to *P. interpunctella* females at low dosages (0.1 μg); however, the attractivity decreased when the dosage was increased up to 1 μg [24]. This phenomenon has also been observed in studies on other insect species. Heptanal promotes the Potato tuber moth’s, *Phthorimaea operculella* (Lepidoptera: Gelechiidae), oviposition at low concentrations and repels oviposition at high concentrations [43].

Behavioral studies require the use of different sites in the laboratory to understand the hierarchical nature of the orienting behavior of the *P. interpunctella*. In both wind tunnel tests, *P. interpunctella* exhibited significant responses to heptanal compared to the control, followed by decanal, octanal, nonanal, and hexanal. However, there was no significant difference in responses to 1-octen-3-ol, hexanoic acid, or acetophenone. Olsson et al. investigated the behavioral response of the *P. interpunctella* to chocolate volatiles in a wind tunnel and found that nonanal was attractive to both female and male *P. interpunctella* [44]. Heptanal and nonanal can influence the oviposition behavior of the Codling moth, *Cydia pomonella* (L.) (Lepidopter: Tortricidae) [45]. Nonanal and decanal, found in maize volatiles, are attractive to the Angoumois grain moth, *Sitotroga cerealella* (Lepidoptera: Gelechiidae) [46]. Additionally, some of these compounds act as attractants for other insects. Hexanal has been shown to significantly attract the Dark-brown chafer, *Holotrichia parallela* (Coleoptera:Scarabaeidae), in field experiments and could be developed into an effective lure for managing this beetle species [47]. Both nonanal and hexanal have demonstrated attractant effects on *S. oryzae* at different concentrations [18]. Moreover, 1-octen-3-ol has also been shown to attract *S. oryzae*, which contrasts with our findings, likely due to differences in feeding and host tendencies.

Cereal compounds can influence the feeding behavior of insects, as well as their oviposition behavior. The choice of oviposition sites is influenced by the volatile compounds produced by host plants, which can have either attractive or repellent effects [48,49]. Not only the oviposition site but also the plant phenology influences insect oviposition [50]. In oviposition experiments, aldehyde compounds exhibited significant attractive effects on *P. interpunctella*, consistent with the findings of Zhao et al. [39]. However, hexanal was found to be the most attractive compound for oviposition. In our experiment, we found that decanal elicited the highest oviposition rate, which might be due to the stronger attraction to hexanal. This discrepancy may be attributed to differences in experimental conditions and oviposition setups. Nonanal has also been reported to show a clear attraction to mated female *P. interpunctella* [51]. Similarly, nonanal and decanal were found to stimulate *P. operculella* females to lay more eggs [52].

## 5. Conclusions

In conclusion, NOPs are an important resource of special VOCs, among which aldehydes (heptanal, decanal, octanal, nonanal, and hexanal) elicited the strongest electrophysiological and oviposition responses in female *P. interpunctella*. These results indicate that heptanal, decanal, octanal, nonanal, and hexanal are the primary chemical cues driving oviposition preferences and therefore represent promising candidates for the development of food-based attractants or monitoring tools.

## Figures and Tables

**Table 1:** Chemical composition of six peanut varieties, including contents of water, protein, fat, carbohydrate, oleic acid, and linoleic acid, determined by peanut quality rapid analyzer.

Peanut Variety	Water (%)	Protein (%)	Fat (%)	Carbohydrate (%)	Oleic Acid (%)	Linoleic Acid (%)
Yuhanghua 1	3.43 ± 0.24	24.91 ± 0.37	53.61 ± 0.42	13.17 ± 0.75	39.57 ± 0.96	33.42 ± 0.34
Yuanza 9847	5.27 ± 0.18	23.94 ± 0.39	51.82 ± 0.27	14.87 ± 0.59	36.87 ± 0.40	37.35 ± 0.51
Wanhua 2	4.21 ± 0.40	25.00 ± 0.22	52.82 ± 0.31	12.47 ± 0.31	39.01 ± 0.43	32.90 ± 0.23
Huayu 16	5.07 ± 0.12	22.55 ± 0.70	51.62 ± 0.18	15.06 ± 0.60	50.06 ± 2.30	30.88 ± 0.20
Fenghua 1	5.35 ± 0.78	23.05 ± 0.28	50.19 ± 0.41	16.73 ± 0.91	58.72 ± 0.89	28.87 ± 0.08
Yuhua 9719	6.55 ± 0.40	24.60 ± 0.16	52.00 ± 0.48	12.35 ± 0.71	37.87 ± 0.59	35.80 ± 0.67

Data in the table are presented as mean ± SE, where *N* = 3 represents the number of replications per peanut variety.

**Table 2:** Identified volatile organic compounds and their quantity (ng/μL) in the headspace of six peanut varieties analyzed by GC−MS.

No.	VOCs	Retention Time (min)	MS Match Range (%)	RI ^a^	RI ^b^	Quantity (ng/μL)					
						Yuhanghua 1	Yuhua 9719	Fenghua 1	Huayu 16	Wanhua 2	Yuanza 9847
1	2,4-dimethyl-heptane	4.455	96–99	793	788	0.84 ± 0.20	—	0.33 ± 0.02	—	—	—
2	butyl acetate	4.486	98–99	791	785	—	—	—	—	0.46 ± 0.06	—
3	Hexanal *	4.560	95–99	797	806	—	—	0.69 ± 0.03	0.64 ± 0.06	0.61 ± 0.05	—
4	4-methyl-octane	5.872	97–99	845	852	—	0.45 ± 0.03	—	—	0.18 ± 0.02	—
5	2,5-dimethyl-heptane	6.056	96–98	796	790	—	—	—	—	—	0.56 ± 0.09
6	1-hexanol *	6.188	95–98	859	860	0.07 ± 0.01 b	0.51 ± 0.05 a	—	—	—	0.42 ± 0.03 a
7	heptanal *	7.366	95–100	883	905	0.44 ± 0.03 a	0.14 ± 0.00 b	—	—	—	0.36 ± 0.03 a
8	(1-methylethyl)-cyclohexane	7.707	98–100	912	915	—	—	—	—	0.20 ± 0.03	0.15 ± 0.01
9	α-pinene	8.272	95–99	957	948	0.19 ± 0.01		0.19 ± 0.01	—	—	—
10	benzaldehyde *	9.365	96–98	973	982	1.24 ± 0.22 a	1.20 ± 0.13 a	—	0.77 ± 0.08 b	—	
11	1,2,3-trimethyl- benzene	9.512	98–99	1011	1020	—	—	—	—	—	0.10 ± 0.01
12	1-methyl-2-propyl-cyclohexane	10.159	98	1026	1040	—	—	0.28 ± 0.07	—	—	—
13	1-octen-3-ol *	10.361	97–100	964	969	1.02 ± 0.10 ab	1.32 ± 0.40 a	0.27 ± 0.06 b	—	—	0.67 ± 0.06 ab
14	2,2,4,6,6-pentamethyl- heptane	10.447	99–100	977	981	0.89 ± 0.06	3.07 ± 0.56	—	—	—	—
15	decane *	11.069	97–100	1007	1015	0.71 ± 0.08 ab	0.60 ± 0.19 ab	0.45 ± 0.05 b	1.17 ± 0.23 a	0.85 ± 0.12 ab	0.57 ± 0.10 ab
16	hexanoic acid, ethyl ester	11.178	98–100	983	984	0.57 ± 0.05	0.52 ± 0.03	—	—	—	—
17	octanal *	11.242	98–100	1000	1007	—	0.35 ± 0.03 a	—	—	0.27 ± 0.03 a	0.31 ± 0.04 a
18	limonene *	12.013	99–100	1011	1018	0.66 ± 0.08 b	0.71 ± 0.09 b	1.49 ± 0.25 a	0.20 ± 0.02 b	0.58 ± 0.04 b	1.52 ± 0.17 a
19	2-ethyl-1-hexanol	12.345	98–100	986	995	—	—	—	0.48 ± 0.09	—	0.13 ± 0.01
20	phenylacetaldehyde *	12.749	97–99	1038	1043	—	—	0.13 ± 0.01 b	—	0.32 ± 0.04 a	0.31 ± 0.06 ab
21	acetophenone *	13.029	97–100	1069	1078	2.36 ± 0.36 a	0.65 ± 0.09 b	—	2.12 ± 0.27 a	—	—
22	undecane	13.316	95–97	1108	1115	—	—	0.13 ± 0.01	—	—	0.31 ± 0.06
23	hexanoic acid *	13.550	96–99	966	974	—	0.21 ± 0.01 b	—	—	0.36 ± 0.04 ab	0.39 ± 0.05 a
24	2-methyl-decane	14.080	98–99	1059	1051	—	—	—	0.19 ± 0.01	—	—
25	nonanal *	15.158	99–100	1098	1104	0.70 ± 0.13 b	0.55 ± 0.05 b	1.28 ± 0.04 a	0.56 ± 0.14 b	0.35 ± 0.01 b	0.75 ± 0.11 b
26	4-methyl-undecane	15.775	96–99	1145	1150	0.44 ± 0.03	0.24 ± 0.01	—	—	—	—
27	2-decen-1-ol	17.535	95–97	1275	1278	—	—	—	—	0.34 ± 0.02	—
28	dodecane *	18.571	97–99	1209	1214	0.71 ± 0.02 b	1.18 ± 0.06 ab	—	—	1.57 ± 0.29 a	—
29	decanal *	18.854	96–99	1197	1204	0.21 ± 0.05 a	0.14 ± 0.01 ab	—	0.15 ± 0.01 ab	0.19 ± 0.01 a	0.06 ± 0.01 b
30	4-methyl-1-undecene	21.340	97–98	1150	1140	0.16 ± 0.03	—	—	—	—	—
31	tridecane	22.033	95–99	1298	1313	—	—	0.31 ± 0.06	—	1.13 ± 0.05	—
32	dodecanal	23.585	99–100	1395	1402	0.29 ± 0.05	—	0.99 ± 0.07	—	—	—
33	3-methyl- tridecane	24.344	95–98	1339	1349	—	0.29 ± 0.03	—	0.31 ± 0.07	—	—
34	1,1′-(1,4-phenylene)bis-ethanone	26.300	98–99	1375	1378	—	—	2.77 ± 0.20	—	—	—
35	tetradecane *	28.687	98–100	1413	1413	0.55 ± 0.07 b	—	—	1.26 ± 0.09 a	—	0.75 ± 0.05 b
36	hexadecane *	32.728	99–100	1609	1612	1.77 ± 0.15 a	—	0.38 ± 0.03 c	0.36 ± 0.02 c	0.83 ± 0.10 b	0.72 ± 0.04 bc
37	3-methyl-hexadecane	33.594	96–98	1645	1647	0.39 ± 0.05	0.13 ± 0.00	—	—	—	—
38	heptadecane *	35.262	98–100	1695	1711	—	—	0.53 ± 0.02 b	2.01 ± 0.27 a	1.68 ± 0.15 a	0.40 ± 0.04 b
39	6-methyl-octadecane	35.860	97–98	1741	1740	—	—	—	0.70 ± 0.08	—	—
40	nonadecane	36.162	98–100	1910	1910	—	—	0.70 ± 0.09	0.14 ± 0.02	—	—
41	2,6-dimethyl- heptadecane	36.329	96–97	1779	1782	0.41 ± 0.04	—	—	—	—	—
42	2,6,10-trimethyl-heptadecane	36.360	96–99	1877	1882	—	—	0.25 ± 0.05	—	—	0.21 ± 0.07
43	1,2-benzenedicarboxylic acid bis(2-methylpropyl) ester	41.212	97–99	1900	1908	—	—	1.09 ± 0.16	0.33 ± 0.04	—	—
44	1,2-benzenedicarboxylic acid, butyl 2-ethylhexyl ester	44.219	98–99	2354	2370	—	—	—	—	1.140 ± 0.21	0.66 ± 0.08

Data in the table are shown as mean ± SE, *N* = 3. Lowercase letters in the same row indicate significant differences among varieties (one-way ANOVA, Tukey’s test, *p* < 0.05). “—” denotes no detection; “*” shows the VOCs measured in electrophysiological and behavioral assays. The MS Match Range (%) represents the range of the mass spectral similarity index between the recorded compound spectra and the library reference spectra, encompassing all tested peanut varieties in which the compound was detected. RI ^a^ was determined on a HP-5 MS column using a homologous series of n-alkanes (C8–C40). RI ^b^ was obtained from the literature for a compound whose identity was established based on a comparison of the retention time and mass spectra data with the authentic standard.

## Data Availability

The original contributions presented in the study are included in the article, further inquiries can be directed to the corresponding author.
